# Use of bovine recombinant prion protein and real-time quaking-induced conversion to detect cattle transmissible mink encephalopathy prions and discriminate classical and atypical L- and H-Type bovine spongiform encephalopathy

**DOI:** 10.1371/journal.pone.0172391

**Published:** 2017-02-22

**Authors:** Soyoun Hwang, Justin J. Greenlee, Eric M. Nicholson

**Affiliations:** Virus and Prion Research Unit, National Animal Disease Center, Agricultural Research Service, United States Department of Agriculture, Ames, IA, United States of America; Creighton University, UNITED STATES

## Abstract

Prions are amyloid-forming proteins that cause transmissible spongiform encephalopathies through a process involving conversion from the normal cellular prion protein to the pathogenic misfolded conformation (PrP^Sc^). This conversion has been used for *in vitro* assays including serial protein misfolding amplification and real-time quaking induced conversion (RT-QuIC). RT-QuIC can be used for the detection of prions in a variety of biological tissues from humans and animals. Extensive work has been done to demonstrate that RT-QuIC is a rapid, specific, and highly sensitive prion detection assay. RT-QuIC uses recombinant prion protein to detect minute amounts of PrP^Sc^. RT-QuIC has been successfully used to detect PrP^Sc^ from different prion diseases with a variety of substrates including hamster, human, sheep, bank vole, bovine and chimeric forms of prion protein. However, recombinant bovine prion protein has not been used to detect transmissible mink encephalopathy (TME) or to differentiate types of bovine spongiform encephalopathy (BSE) in samples from cattle. We evaluated whether PrP^Sc^ from TME and BSE infected cattle can be detected with RT-QuIC using recombinant bovine prion proteins, and optimized the reaction conditions to specifically detect cattle TME and to discriminate between classical and atypical BSE by conversion efficiency. We also found that substrate composed of the disease associated E211K mutant protein can be effective for the detection of TME in cattle and that wild type prion protein appears to be a practical substrate to discriminate between the different types of BSEs.

## Introduction

Prion diseases, or transmissible spongiform encephalopathies (TSEs), are a group of fatal neurodegenerative disorders that include Creutzfeldt-Jakob disease (CJD), fatal familial insomnia (FFI), Gerstmann-Sträussler-Scheinker syndrome (GSS) and kuru in humans and bovine spongiform encephalopathy (BSE). They are caused by the misfolding of the cellular prion protein (PrP^C^) into a pathogenic form (PrP^Sc^) that accumulates in the brain and some lymphoid tissues [1–4]. Conformational conversion of PrP^C^ to PrP^Sc^ makes them partially protease resistant.

Since the outbreak of foodborne BSE in the UK, two atypical strains of BSE called H-type BSE (H-BSE) and L-type BSE (L-BSE) have also been identified and named according to the molecular weight difference relative the initially identified UK BSE isolate termed classical-BSE (C-BSE). These atypical BSE forms are believed to arise spontaneously and tend to affect older animals [5]. H- and L-BSE have distinct transmissibility and different regulatory outcomes in the event which makes it important to be detected and differentiated from classical. Western blot can be used to discriminate the classical and atypical BSE forms by the specific molecular weight and glycoform ratios of PrP^Sc^. H-type has a higher molecular weight around 1–2 kDa above C-BSE for the unglycosylated PrP band and L-type BSE has a lower migration around 0.5 kDa below C-BSE [5–9]. Interestingly, L-BSE has phenotypic similarities with cattle transmissible mink encephalopathy (TME) [10–12]. TME is a progressive neurodegenerative disease that was identified in ranched mink. This disease is still poorly understood in terms of the origin of the infection, but it is speculated to have been derived from an unknown TSE in cattle [13, 14].

Detection of PrP^Sc^ can be accomplished by several methods. Traditional assays such as western blot and immunohistochemistry (IHC) are effective for detecting relatively large quantities of PrP^Sc^ present in samples [15, 16], however, they do not have the ability to detect the minute quantities of prions in a sample such as body fluid. Detection of prions using ELISA can be fast, sensitive, and specific, but provides little in the way of discriminatory power. The *in vitro* protein misfolding cyclic amplification (PMCA) assay is very useful for sensitive detection of prions in various samples of different species, but it is time consuming and requires animal derived substrate [17–19]. In contrast, the real-time quaking-induced conversion (RT-QuIC) assay uses recombinant prion protein as a substrate for seeding activity and monitors the fibril formation in real- time via binding of the fluorescent dye thioflavin T (ThT) to amyloid fibrils. The increased ThT fluorescence increase as the amount of amyloid fibril accumulates through conversion of monomeric PrP^C^ to amyloid. The RT-QuIC assay can rapidly detect low levels of prion and has been used to detect human and animal prions in various tissues [20–27]. Performance of various RT-QuIC assays requires appropriate combinations of prion seed and recombinant prion protein substrate. A variety of recombinant prion proteins have been utilized in RT-QuIC assays including hamster [a.a. 23–231, 90–231] [20, 26, 28], human [a.a. 23–231] [26, 28, 29], mouse [a.a. 23–231] [29], bank vole (BV) [a.a. 23–230, 90–230] [29, 30], sheep [a.a. 23–234] [20, 27, 29], elk [a.a. 23–231] [31, 32], a hamster-sheep chimera [hamster a.a. 23–137, sheep a.a. 141–234] [28, 29] and bovine [a.a. 23–231] [33]. TSEs. To investigate the possible use of RT-QuIC for TSE surveillance in cattle we tested the suitability of bovine prion protein (bPrP) as a substrate for cattle TSE detection and discrimination using RT-QuIC reaction including its disease associated mutant form E211K protein that is homologous to the genetic CJD associated human E200K mutation. We have optimized the RT-QuIC reaction conditions for the specific detection of prions in brain samples from cattle with prion disease using bPrP wild type and E211K proteins.

## Materials and methods

### Brain samples from negative control and TME or BSE-infected cattle

The animal experiments were carried out in accordance with the Guide for the Care and Use of Laboratory Animals (Institute of Laboratory Animal Resources, National Academy of Sciences, Washington, DC) and the Guide for the Care and Use of Agricultural Animals in Research and Teaching (Federation of Animal Science Societies, Champaign, IL). The protocol was approved by the Institutional Animal Care and Use Committee at the National Animal Disease Center (protocol numbers: 3636 and 3985). Animals were monitored twice daily to assess overall animal health and to check for the appearance of clinical signs such as aggression, behavior changes, decreased feed intake, loss of body condition, ataxia, prolonged recumbency, or inability to rise. All inoculated animals developed clinical signs specifically described in Table 1 and no animals died prior to the experimental endpoint. To ensure a humane endpoint, animals were euthanized if they demonstrated clinical signs of prolonged anorexia (>24 hours) or recumbency (>12 hours). Animals exhibiting behavioral or postural changes that suggested a TSE were examined by a veterinarian. Upon veterinary confirmation of unequivocal clinical signs of TSE, affected cattle were killed with an intravenous injection of pentobarbital sodium according to the manufacturer’s directions and necropsied. Any other concurrent diseases were treated under the direction of a veterinarian with drugs to alleviate pain or distress.

**Table 1:** Animal donor information. Brain tissues collected from cattle that were normal or clinically ill with BSE were used to prepare homogenates.

Animal Number	Inoculated TSE	Souce of inoculum	Incubation time (days)	Signs	Clinical description
52AA	TME	3^rd^ cattle passage	579	+	progressively unaware of surroundings, difficulty rising, stagerred when walking
6895	L-type BSE	French Case	475	+	presented as downer animal without prior indication of clinical signs
6930	L-type BSE	French Case	462	+	loss of body condition, low head carriage, mentally dull
6880	H-type BSE	U.S. BSE (2006)	490	+	decreased feed intake, slow to rise, unstable on feet when walking
80	H-type BSE	U.S. BSE (2006)	543	+	listless, low head carriage, head pressing, forelimb ataxia, circling in pen
83	H-type BSE	U.S. BSE (2006)	294	+	head pressing, reluctant to rise, ataxic, repetative chewing behaviors
4	H-type BSE	U.S. BSE (2006)	385	+	abnormal stance, excessive chewing and salivation, head tremor
6836	classical	U.S. 2003 (WA)	639	+	sawhorse stance, irratable when encouraged to walk, fell down while walking
6992	classical	U.S. 2003 (WA)	638	+	sawhorse stance, increased startle response, goose-stepping gate

Samples of brainstem at the level of the obex were obtained from sham inoculated negative controls and cattle experimentally infected with TME or H-type, L-type, or classical BSE. Results of western blots and microscopic analysis of samples from the brains of cattle infected with TME are described elsewhere [34–36]. Original sources of the BSE inocula were as follows: French L-type BSE [37], U.S. classical and H-type BSE [38]. Brain homogenates (10%, w/v) from normal cow and cattle infected with TME and different BSE strains were prepared as whole homogenates of brainstem sections by bead homogenization with 1.0 mm silica beads in 1X Dulbecco’s PBS pH 7.4. Samples were centrifuged briefly (1000g for 2 min) and supernatants stored at -80°C in aliquots. The negative control animal was sham inoculated with 1 ml of 10% brain homogenate from a negative control animal and housed separately from the other cattle in this study. Table 1 shows all animal donor information including TSE isolate inoculated, source of inoculum, incubation times, and description of clinical signs.

### Western blotting of cattle brain homogenates (BH)

Western blot analyses was performed on brain tissue collected from one non-inoculated, one TME-infected, and eight BSE-infected cattle: two classified as C-type, four as H-type, and two as L -type. Each sample was separated by SDS-PAGE on 12% polyacrylamide minigels (Invitrogen) and then transferred onto polyvinylidene difluoride (PVDF) membranes (Millipore, Billerica, MA) for 45 min at 30 V. The membranes were blocked with 3% bovine serum albumin (BSA) in Tris-buffered saline (TBS) for 1 h and incubated at 4°C overnight with mouse anti-PrP monoclonal antibody 6H4 at a 1:10,000 dilution (0.1 μg/ml) as the primary antibody. Then, a biotinylated sheep anti-mouse secondary antibody at 0.05 μg/ml and a streptavidin–horseradish peroxidase (HRP) conjugate, were used in conjunction with a chemiluminescent detection system (Pierce ECL plus, Thermo Fisher) and visualized on an imaging system capable of detecting luminescence.

### EIA of brain homogenates from cattle

The relative amount of misfolded protein in brain homogenates from the TME and BSE infected cattle were determined using a IDEXX HerdChek BSE EIA kit in the absence of proteinase K digestion. EIA was performed as described by the manufacturer. The cutoff value was determined by the negative control sample provided by the manufacturer and the optical density value was around 0.15 ± 0.05. If the optical density value is over 0.3, the samples would be considered positive. All brain samples were normalized before analysis by RT-QuIC by diluting to an O.D. around 1.0.

### Recombinant prion protein production and purification

*E*. *coli* (BL21(λDE3)) was transformed with the pET28a vector containing the wild-type bovine PrP gene (amino acids 25–241; GenBank accession number: DQ875147.1) and the bovine prion protein (wild type or E211K)was expressed and purified as described by Vrentas *et al* [39]. Briefly, *E*. *coli* strains (BL21(λDE3)) containing the wild type bovine prion gene and E211K were cultured in separate flasks of LB medium (5 ml) supplemented with kanamycin (50 μg/ml). The cells were grown for 16 h at 37°C with shaking at 250 rpm. Each culture (2 mL) was then transferred to fresh 1 L of LB medium containing the same concentrations of antibiotics. The cultures were grown at 37°C, harvested, and the cell pellet (3–4 g) was suspended and lysed in buffer A (0.15 M NaCl, 2 mM EDTA, 10 mM Tris–HCl (pH 7.4), 0.1% Triton X-100), and inclusion bodies (IB) were washed in buffers B-1 (20 mM Tris–HCl (pH 7.4), 0.15 M NaCl, 0.5% Triton X-100), B-2 (20 mM Tris–HCl (pH 7.4), 2 M NaCl), and B-3 (0.15 M NaCl, 20 mM Tris–HCl (pH 7.4), 2 M urea). Prepared inclusion bodies (IBs) were mixed with 60–80 ml buffer including 6 M guanidine-HCl, 10 mM Tris-HCl (pH 8), 0.1 M Na_2_HPO_4_, 0.5% Triton X-100 and incubated at room temperature for 1 h before overnight incubation at 4°C on a rocker. Wild type and E211K were isolated using Ni-NTA (Nickel) resin (Qiagen, #30210) and washed with IMAC buffer (8 M urea, 0.1 M Na_2_HPO_4_, 10 mM Tris–HCl, pH adjusted to 8) and eluted with buffer L (8 M urea, 10 mM Tris–HCl, 0.3 M imidazole, pH adjusted to 8). The eluate (10 ml) was collected in 1 ml fractions by gravity flow. The individual fractions were analyzed by SDS-PAGE. All eluted pooled fractions were diluted to a final protein concentration of 0.5 mg/ml and dialyzed overnight into IMAC buffer containing 8 M urea, 0.1 M Na_2_HPO_4_, and 10 mM Tris-HCl adjusted to pH 8. The second dialysis was performed overnight into 10 mM potassium phosphate buffer adjusted to pH 7. The concentration of pooled protein eluent was measured by UV and calculated from the absorbance at 280 nm using an extinction coefficient of 63,495 M^-1^cm^-1^ as calculated for wild type and E211K proteins.

### RT-QuIC protocol

RT-QuIC reactions were performed as previously described [27–30, 40, 41]. The reaction mix was composed of 10 mM phosphate buffer (pH 7.4), 100 mM to 500 mM NaCl, 0.1 mg/ml recombinant bovine prion protein wild type or E211K protein, 10 μM thioflavin T (ThT), 1 mM ethylenediaminetetraacetic acid tetrasodium salt (EDTA), and 0.001% or 0.002% SDS. Aliquots of the reaction mix (98 μL) were loaded into each well of a black 96-well plate with a clear bottom (Nunc, Thermo Fisher Scientific) and seeded with 2 μL of brain homogenate dilution. The plate was then sealed with plate sealer film and incubated at 42°C in a BMG FLUOstar Omega plate reader with cycles of 15 min shaking (700 rpm double orbital) and 15 min rest for 100 h. ThT fluorescence measurements (excitation, 460 nm; emission 480 nm, bottom read, 20 flashes per well, manual gain 1400) were taken every 45 min.

To optimize the conditions of the RT-QuIC reaction, assays using brain samples from negative control or TSE infected cattle were performed with wild type and E211K substrates with and without SDS and at different NaCl concentrations (100, 200, 300, 400, 500 mM). Once optimized, RT-QuIC was performed using quadruplicates of two dilutions (10^−4^–10^−6^) of normal and infected cattle brain homogenates. All reactions for each dilution and each sample were performed in quadruplicates in two independent RT-QuIC assays for a total of 8 replicates.

ThT fluorescence data are displayed as the average ThT fluorescence of four technical replicates for each time point and, to be considered positive, the ThT fluorescence of at least two replicate reactions must be positive. The positive threshold was calculated as the mean value of normal cattle brain homogenates plus 10 standard deviations. Previously described criteria were applied for classification of positive samples of RT-QuIC [27, 42, 43].

### Brain homogenate preparations

BHs (10% w/v) were prepared as previously described [20] and stored at -80°C. For RT-QuIC analysis, BHs were serially diluted in 0.1% SDS/N2 or 0.05% SDS/N2 (Gibco)/PBS as previously reported [24].

## Results

### Western blotting and quantitation of PrP^Sc^ by EIA in brain samples from TME and BSE infected cattle

**Fig 1:**
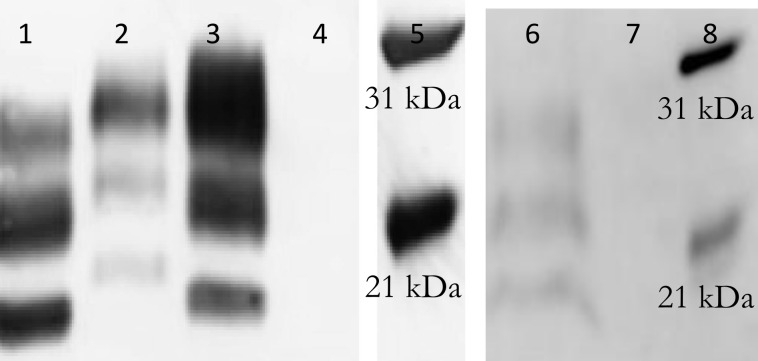
Western blot of a representative brain sample of each BSE strain and TME in cattle. Brainstem samples were characterized by western blotting (mAb 6H4). Lanes from left: 1. French L-type (#6895, 2 mg);; 2. U.S. H-type (#80, 2 mg); 3. Classical (#6836, 1 mg); 4.Negative Control (#6969, 2 mg); 5. Biotinylated protein marker; 6. Bovine TME (Animal #52AA, 2 mg); 7. Negative Control (#6969, 2mg); 8. Biotinylated protein marker.

The brainstem samples (obex) were collected from cattle affected by TME or C-, L-, or H-BSE. To confirm that strain specific properties of each inoculum were transmitted to the experimental animals, brainstem tissue from selected animals was characterized by western blot using the 6H4 antibody. Most of these brain samples have been studied previously, so only a representative brain sample of each strain was tested. Western blots demonstrated PrP^Sc^ with the typical band pattern profiles of C-, L-, and H-BSE (Fig 1).

### Brain samples from TME infected-cattle seed the conversion of both wild type and E211K bovine recombinant prion proteins

**Fig 2:**
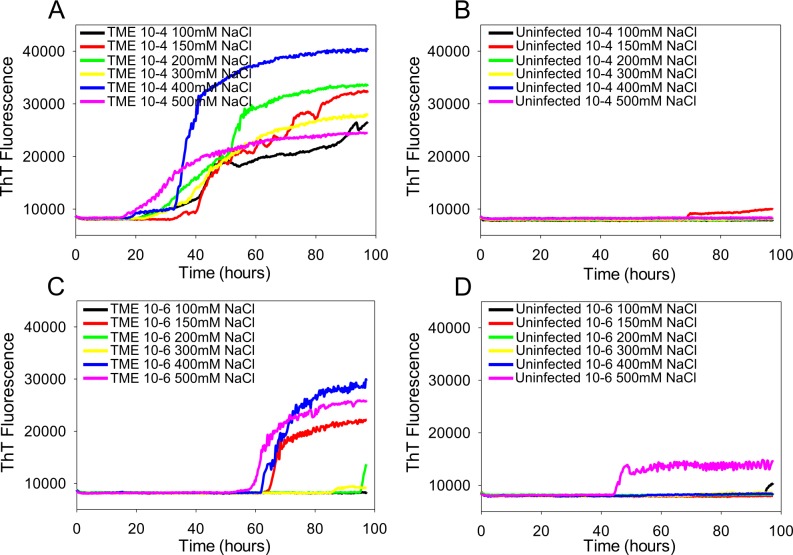
RT-QuIC sodium chloride titration for TME-infected and uninfected cattle brain samples. RT-QuIC reactions were seeded with 10^−4^ (A, B) and 10^−6^ dilution (C, D) of TME-infected and uninfected cattle brain homogenates using a range of NaCl concentrations (100–500 mM) with the full-length bPrP wild type (aa 25–241) as substrate. Data are presented as mean ThT fluorescence of 8 reactions conducted as 2 repeats of 4 reactions. The positive threshold was calculate as ~10,000 relative fluorescence units of normal cattle brain homogenates.

**Fig 3:**
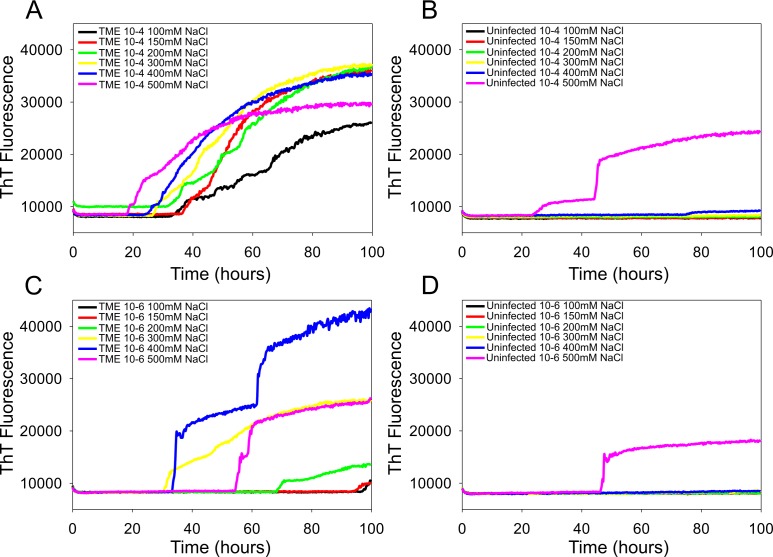
RT-QuIC sodium chloride titration for TME-infected and uninfected cattle brain samples. RT-QuIC reactions were seeded with 10^−4^ (A, B) and 10^−6^ dilution (C, D) of TME-infected and uninfected cattle brain homogenates using a range of NaCl concentrations (100–500 mM) with the full-length bPrP E211K protein (aa 25–241) as substrate. Data are presented as mean ThT fluorescence of 8 reactions conducted as 2 repeats of 4 reactions. The positive threshold was calculate as ~10,000 relative fluorescence units of normal cattle brain homogenates.

To investigate if RT-QuIC can be used to detect prion seeding activity from TME infected cattle brain by using bovine recombinant prion protein substrates, we used infected and normal brain homogenates with full length bovine rPrP [a.a. 25–241] substrate including wild type and the mutant E211K protein and the reaction conditions (0.1 mg/ml rPrP final concentration, 300 mM NaCl and 0.001% SDS). An increase in ThT fluorescence was observed within 30 h incubation in each quadruplicate reaction seeded with 10^−4^ dilution of 10% w/v TME brain homogenate (Fig 2 and Fig 3). Reaction seeded with more diluted 10^−6^ dilution of 10% w/v TME brain homogenate at 60 h but no increase in fluorescence was observed in normal brain homogenates reactions at any dilution except with high NaCl concentrated samples.

### Optimization of RT-QuIC for the detection of cattle TSE prion seeding activity

To optimize the sensitivity of RT-QuIC reactions for the detection of TME prion seeding activity, we compared the effects of SDS (0.001% vs. 0.002%), NaCl (100–500 mM) concentration, and different bPrP substrates (WT (Fig 2) vs. E211K (Fig 3)). The effect of NaCl on the seeding activity with TME prion was not profound when bPrP wild type was used (Fig 2). However, when the E211K protein was used, the effect of NaCl on the seeding activity with TME prion was slightly different (Fig 3). As can be seen in Fig 3, an assay with reaction mixture containing 500 mM NaCl and bPrP-E211K protein as a substrate shows a slightly reduced lag time and higher ThT fluorescence in a reaction seeded with positive TME brain homogenate indicating a higher conversion efficiency, but it also increases ThT fluorescence with normal brain. When using lower NaCl concentrations such as 100 mM to 200 mM, there is increased ThT fluorescence with a longer lag phase without spontaneous fibril formation. With 300 and 400 mM NaCl concentrations, the seeding activity is readily detected with no evidence of spontaneous fibril formation when seeded with normal brains. Overall, our data indicate that a slightly reduced lag time for detectable seeding activity in cattle brain samples was observed when we used 500 mM NaCl, and the effect of NaCl between 200 mM and 400 mM on the lag time was not significant.

**Fig 4:**
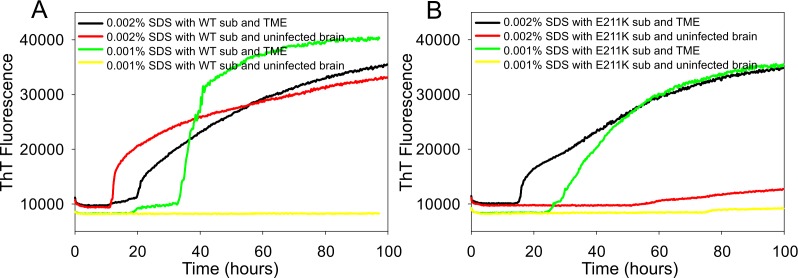
**Comparison of SDS dependency of RT-QuIC reactions using full-length (A) bPrP wild type or (B) bPrP E211K as the substrate.** RT-QuIC reactions were seeded with 10^−4^ dilutions of TME infected cattle and negative control (uninfected cattle) brain homogenates using either full-length bPrP wild type or E211K protein (aa 25–241) as substrates with the addition of 0.001% or 0.002% SDS (final concentration). Data are presented as mean ThT fluorescence of 8 reactions conducted as 2 repeats of 4 reactions. The positive threshold was calculate as ~10,000 relative fluorescence units of normal cattle brain homogenates.

The effect of SDS on the seeding activity with TME prion was also tested. Higher SDS concentration reduced the lag phase for prion seeding activity for both substrates by 10 h (Fig 4A). However, higher SDS concentration also triggered spontaneous fibril formation in bPrP when seeded with brain sample from negative control cattle. Interestingly, RT-QuIC using rPrP-E211K protein substrate did not form fibrils after seeding with normal brain (Fig 4B).

**Fig 5:**
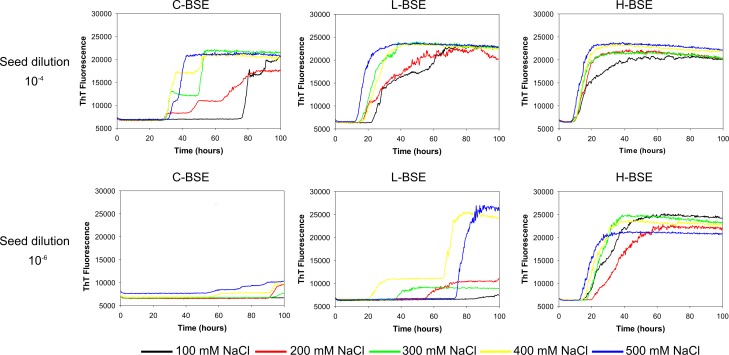
RT-QuIC sodium chloride titration for different types of BSE-infected cattle brain samples. RT-QuIC reactions were seeded with 10^−4^ and 10^−6^ dilution of C-, L, and H-BSE-infected cattle brain homogenates using a range of NaCl concentrations (100–500 mM) with the full-length bPrP wild type (aa 25–241) as substrate. Data are presented as mean ThT fluorescence of 8 reactions conducted as 2 repeats of 4 reactions. The positive threshold was calculate as ~10,000 relative fluorescence units of normal cattle brain homogenates.

The effect of NaCl on the seeding activity with different BSE isolates was also evaluated with bPrP wild type substrate (Fig 5) at an SDS concentration of 0.001% the effect of NaCl concentration over a range from 100 mM to 500 mM. Concentrations of NaCl greater than 300 mM resulted in higher levels of fluorescence in the conversion curves and a reduced lag time for L-type BSE. However, the lag time was essentially unchanged for classical and H-type BSE. Based on these results an SDS concentration of 0.001% and 300 mM NaCl was deemed suitable for all BSE isolates analyzed here.

### Detection of H-BSE, L-BSE, and C-BSE prion seeding activity using bPrP substrates

**Fig 6:**
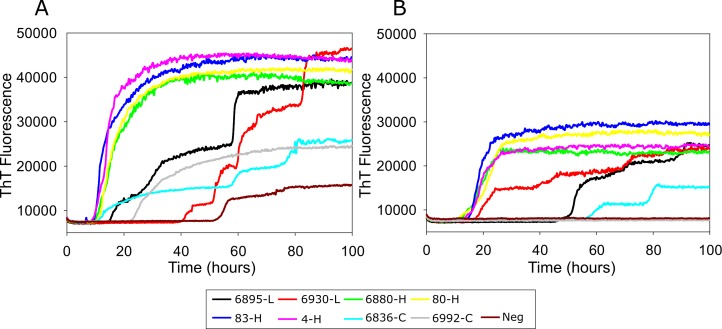
**RT-QuIC detection of C-, L-, and H-BSE prion seeding activity using bovine prion proteins (25–241) wild type (A) and its disease associated form E211K protein (B).** RT-QuIC reaction mixtures were seeded with 10^−4^ dilutions of brain tissues from uninfected, C-BSE-affected (cyan and grey lines), L-BSE-affected (red and black lines) and H-BSE-affected (pink, blue, yellow, green lines) cattle. A final SDS concentration of 0.001% in combination with 300 mM NaCl was used with the substrates. Data are presented as mean ThT fluorescence of 8 reactions conducted as 2 repeats of 4 reactions. The positive threshold was calculate as ~10,000 relative fluorescence units of normal cattle brain homogenates.

We used the bovine substrates including wild type and disease associated mutant E211K to detect different strains of BSE (H-, L-, and C-BSE). Reaction mixtures seeded with 10^−4^ dilutions of brain tissue samples from four cattle infected with H-type BSE, two cattle with L-type BSE, and two cattle with classical BSE (Fig 6). Assays seeded with H-BSE showed rapid increases in ThT fluorescence within 10 h. Conversely, reaction mixtures seeded with the same dilution of brain samples from C-BSE infected cattle gave slightly longer lag phases and lower ThT fluorescence than the reaction mixtures with H-BSE. L-BSE seeded reaction mixtures gave much longer lag phases and similar ThT fluorescence level compared to H-BSE reactions. Replicate reactions on brain samples from negative control cattle were negative for about 50 h in wild type substrate (Fig 6A) and 90 h in E211K substrate (Fig 6B). Recently shown RT-QuIC reaction with bank vole substrate showed a similar ability to discriminate different types of BSEs except that C-BSE seeded reaction had longest lag phases out of three types of BSE reactions [29]. These results indicate that H-BSE was more rapidly detected than either C- or L-BSE using the same concentration of brain homogenate from clinically affected cattle. Compared to the reaction with wild type substrate, reaction with E211K substrate responded in a similar way to H-BSE but with lower fluorescence emission levels for all samples and lower sensitivity and longer lag phase for C-BSE. This result indicates that RT-QuIC using wild type substrates can distinguish different strains of BSE and comparing seeding activities with the two substrates will allow us to distinguish C-BSE from atypical BSEs.

## Discussion and conclusions

Recombinant bovine prion protein, including its disease associated mutant form E211K were used as an RT-QuIC substrate to demonstrate that bovine proteins can be seeded in RT-QuIC reactions by brain samples from cattle infected with TME or different types of BSE. We initially used previously described reaction conditions for detecting seeding activity associated with BSE in cattle using bank vole substrate and found RT-QuIC can specifically detect TME and BSE prion seeds in samples from the brains of infected cattle. Cattle TME was used for the optimization of RT-QuIC reaction since it is similar to and perhaps may even be the same as L-BSE [10–12]. Our data indicate that using full-length bovine protein substrate with 0.001% SDS and 300 mM NaCl provided optimal amplification of prion seeding activity in cattle brain samples within 50 h incubation period for TME and within 30 h for BSE. Our data also confirmed that brain samples from TME infected cattle can seed the conversion reaction using E211K mutant protein indicating that the mutant protein appears largely interchangeable with the wild type for RT-QuIC for detecting TME in cattle. E211K protein is a disease associated form that is homologous to the human E200K mutation found in patients with Creutzfeldt-Jakob disease. This genetically associated BSE was diagnosed in the U.S. in 2006 [44] as an H-type BSE. The E211K index animal had a polymorphism that resulted in a glutamic acid to lysine substitution at residue 211 [45]. Until this case, no example of inherited prion disease analogous to CJD in human has been identified in non-human species [45–47].

RT-QuIC has been used for sensitive and specific detection of prion diseases for humans and animals [20–22, 24–26, 48]. Recently, Orru *et al*. showed RT-QuIC can be used for detection and discrimination of L-BSE and C-BSE prions by using chimeric form of prion protein substrate (hamster-Sheep) and other substrates including hamster and human protein substrates [28]. Masujin *et al*. also tested if all three types of BSE including C-, H-, and L-BSE prions can be detected and discriminated by various recombinant protein substrates including full-length chimeric hamster-sheep prion proteins, N-terminally truncated hamster prion protein [90–231][29], full-length sheep protein, and full-length bank vole protein (a.a. 23–230). The later work demonstrated a means to detect and discriminate clearly each of three major bovine prion strains by running simultaneously with bank vole and sheep substrate in the plate [29]. When they used recombinant bank vole prion protein as a substrate, all types of BSE turn out positive, but H- and L-BSE seeded reaction products were not distinguished from one another by RT-QuIC alone. However, it appears bovine protein substrate can discriminate the major bovine prion strains as can be seen in Fig 5. Assays of samples from cattle infected with H-BSE exhibited increased ThT fluorescence within 10 h, but samples from cattle infected with C-BSE showed slightly longer lag phases with lower overall ThT fluorescence. RT-QuIC assays performed on samples from catle infected with L-BSE gave similar lag phases to C-BSE but slowly increased ThT fluorescence level compared to short lag phase before the observed fluorescence increase for H-BSE. Even though bPrP wild type can be used to discriminate different BSE strains by itself, running reactions with both bPrP wild type and E211K protein at the same time would discriminate more clearly between atypical BSEs and C-BSE since E211K protein is rarely seeded by C-BSE.

It is important to note that there are differences in seeding activity between wild type bovine protein and its mutant E211K protein. First, a notable difference between using these two substrates was seen in the reaction with different concentration of SDS. For wild type, we observed a spontaneous increase in ThT fluorescence when the reaction mixture contained 0.002% SDS seeded with a brain sample from a negative control animal. On the contrary, the reaction mixture with substrate E211K protein did not produce ThT fluorescence for seed from negative control brain in the presence of 0.002% SDS and high salt concentration. Another notable difference is when brain samples from BSE- infected cattle were used for seeding these substrates, wild type protein could detect all different types of samples by different lag phase curves and different levels of ThT fluorescence. However, E211K protein was only weakly seeded with classical BSE samples. As can be seen in the Fig 5, the assay seeded with animal 6836 (C-BSE) exhibited a long lag phase and had fluorescence only slightly above the pre-defined positive threshold. Also, the reaction with animal 6992 (C-BSE) did not amplify at all. These results indicate that wild type and E211K protein may adopt a different conformation under given reaction conditions so that they have different fibril formation mechanisms. As pointed by Corsaro et al, human prion protein and its mutant E200K, which is analogous to E211K, showed differences in fibril formation and gain of toxicity characterized by physicochemical characteristics [23]. In spite of essentially identical NMR structures of wild type and E200K, protein surface charge alteration may change the ability of PrP to interact with cell membrane proteins or chaperones [49].

In summary, this is the first published report of RT-QuIC using bovine recombinant prion protein as a substrate for the amplification of bovine TSEs (TME, C-BSE, H-BSE, and L-BSE). In particular, the wild type bovine protein is a practical substrate to discriminate between different BSE strains using the RT-QuIC assay.

## References

[1] Prusiner SB. Prions. Proc Natl Acad Sci U S A. 1998;95(23):13363–83. PubMed Central PMCID: PMC33918. 9811807 10.1073/pnas.95.23.13363 PMC33918

[2] Collinge J. Prion diseases of humans and animals: their causes and molecular basis. Annu Rev Neurosci. 2001;24:519–50. 10.1146/annurev.neuro.24.1.519 11283320

[3] Weissmann C. The Ninth Datta Lecture. Molecular biology of transmissible spongiform encephalopathies. FEBS letters. 1996;389(1):3–11. 8682199 10.1016/0014-5793(96)00610-2

[4] Caughey B, Chesebro B. Transmissible spongiform encephalopathies and prion protein interconversions. Advances in virus research. 2001;56:277–311. 11450303 10.1016/s0065-3527(01)56031-5

[5] Biacabe AG, Laplanche JL, Ryder S, Baron T. Distinct molecular phenotypes in bovine prion diseases. EMBO Rep. 2004;5(1):110–5. PubMed Central PMCID: PMC1298965. 10.1038/sj.embor.7400054 14710195 PMC1298965

[6] Beringue V, Bencsik A, Le Dur A, Reine F, Lai TL, Chenais N, et al Isolation from cattle of a prion strain distinct from that causing bovine spongiform encephalopathy. PLoS Pathog. 2006;2(10):e112 PubMed Central PMCID: PMC1617128. 10.1371/journal.ppat.0020112 17054396 PMC1617128

[7] Casalone C, Zanusso G, Acutis P, Ferrari S, Capucci L, Tagliavini F, et al Identification of a second bovine amyloidotic spongiform encephalopathy: molecular similarities with sporadic Creutzfeldt-Jakob disease. Proc Natl Acad Sci U S A. 2004;101(9):3065–70. PubMed Central PMCID: PMC365745. 10.1073/pnas.0305777101 14970340 PMC365745

[8] Buschmann A, Biacabe AG, Ziegler U, Bencsik A, Madec JY, Erhardt G, et al Atypical scrapie cases in Germany and France are identified by discrepant reaction patterns in BSE rapid tests. Journal of virological methods. 2004;117(1):27–36. 10.1016/j.jviromet.2003.11.017 15019257

[9] Buschmann A, Gretzschel A, Biacabe AG, Schiebel K, Corona C, Hoffmann C, et al Atypical BSE in Germany—proof of transmissibility and biochemical characterization. Veterinary microbiology. 2006;117(2–4):103–16. 10.1016/j.vetmic.2006.06.016 16916588

[10] Comoy EE, Mikol J, Ruchoux MM, Durand V, Luccantoni-Freire S, Dehen C, et al Evaluation of the zoonotic potential of transmissible mink encephalopathy. Pathogens. 2013;2(3):520–32. PubMed Central PMCID: PMC4235697. 10.3390/pathogens2030520 25437205 PMC4235697

[11] Baron T, Bencsik A, Biacabe AG, Morignat E, Bessen RA. Phenotypic similarity of transmissible mink encephalopathy in cattle and L-type bovine spongiform encephalopathy in a mouse model. Emerging infectious diseases. 2007;13(12):1887–94. PubMed Central PMCID: PMC2876762. 10.3201/eid1312.070635 18258040 PMC2876762

[12] Vrentas CE, Greenlee JJ, Baron T, Caramelli M, Czub S, Nicholson EM. Stability properties of PrP(Sc) from cattle with experimental transmissible spongiform encephalopathies: use of a rapid whole homogenate, protease-free assay. BMC Vet Res. 2013;9:167 PubMed Central PMCID: PMC3751458. 10.1186/1746-6148-9-167 23945217 PMC3751458

[13] Marsh RF, Bessen RA, Lehmann S, Hartsough GR. Epidemiological and experimental studies on a new incident of transmissible mink encephalopathy. The Journal of general virology. 1991;72 (Pt 3):589–94. 1826023 10.1099/0022-1317-72-3-589

[14] Marsh RF, Burger D, Eckroade R, Zu Rhein GM, Hanson RP. A preliminary report on the experimental host range of the transmissible mink encephalopathy agent. The Journal of infectious diseases. 1969;120(6):713–9. 4986938 10.1093/infdis/120.6.713

[15] Van Everbroeck B, Pals P, Martin JJ, Cras P. Antigen retrieval in prion protein immunohistochemistry. The journal of histochemistry and cytochemistry: official journal of the Histochemistry Society. 1999;47(11):1465–70. 10544219 10.1177/002215549904701112

[16] Peretz D, Williamson RA, Matsunaga Y, Serban H, Pinilla C, Bastidas RB, et al A conformational transition at the N terminus of the prion protein features in formation of the scrapie isoform. J Mol Biol. 1997;273(3):614–22. 10.1006/jmbi.1997.1328 9356250

[17] Soto C, Saborio GP, Anderes L. Cyclic amplification of protein misfolding: application to prion-related disorders and beyond. Trends in neurosciences. 2002;25(8):390–4. 10.1016/s0166-2236(02)02195-1 12127750

[18] Soto C, Anderes L, Suardi S, Cardone F, Castilla J, Frossard MJ, et al Pre-symptomatic detection of prions by cyclic amplification of protein misfolding. FEBS letters. 2005;579(3):638–42. 10.1016/j.febslet.2004.12.035 15670821

[19] Saa P, Castilla J, Soto C. Cyclic amplification of protein misfolding and aggregation. Methods in molecular biology. 2005;299:53–65. 15980595 10.1385/1-59259-874-9:053

[20] Wilham JM, Orru CD, Bessen RA, Atarashi R, Sano K, Race B, et al Rapid end-point quantitation of prion seeding activity with sensitivity comparable to bioassays. PLoS Pathog. 2010;6(12):e1001217 PubMed Central PMCID: PMC2996325. 10.1371/journal.ppat.1001217 21152012 PMC2996325

[21] Atarashi R, Sano K, Satoh K, Nishida N. Real-time quaking-induced conversion: a highly sensitive assay for prion detection. Prion. 2011;5(3):150–3. PubMed Central PMCID: PMC3226039. 10.4161/pri.5.3.16893 21778820 PMC3226039

[22] Atarashi R, Satoh K, Sano K, Fuse T, Yamaguchi N, Ishibashi D, et al Ultrasensitive human prion detection in cerebrospinal fluid by real-time quaking-induced conversion. Nature medicine. 2011;17(2):175–8. 10.1038/nm.2294 21278748

[23] Corsaro A, Thellung S, Bucciarelli T, Scotti L, Chiovitti K, Villa V, et al High hydrophobic amino acid exposure is responsible of the neurotoxic effects induced by E200K or D202N disease-related mutations of the human prion protein. The international journal of biochemistry & cell biology. 2011;43(3):372–82. 21094273 10.1016/j.biocel.2010.11.007

[24] McGuire LI, Peden AH, Orru CD, Wilham JM, Appleford NE, Mallinson G, et al Real time quaking-induced conversion analysis of cerebrospinal fluid in sporadic Creutzfeldt-Jakob disease. Ann Neurol. 2012;72(2):278–85. PubMed Central PMCID: PMC3458796. 10.1002/ana.23589 22926858 PMC3458796

[25] Orru CD, Wilham JM, Vascellari S, Hughson AG, Caughey B. New generation QuIC assays for prion seeding activity. Prion. 2012;6(2):147–52. 10.4161/pri.19430 22421206 PMC7082091

[26] Peden AH, McGuire LI, Appleford NE, Mallinson G, Wilham JM, Orru CD, et al Sensitive and specific detection of sporadic Creutzfeldt-Jakob disease brain prion protein using real-time quaking-induced conversion. The Journal of general virology. 2012;93(Pt 2):438–49. PubMed Central PMCID: PMC3352348. 10.1099/vir.0.033365-0 22031526 PMC3352348

[27] Dassanayake RP, Orru CD, Hughson AG, Caughey B, Graca T, Zhuang D, et al Sensitive and specific detection of classical scrapie prions in the brains of goats by real-time quaking-induced conversion. The Journal of general virology. 2016;97(3):803–12. 10.1099/jgv.0.000367 26653410 PMC5972304

[28] Orru CD, Favole A, Corona C, Mazza M, Manca M, Groveman BR, et al Detection and discrimination of classical and atypical L-type bovine spongiform encephalopathy by real-time quaking-induced conversion. Journal of clinical microbiology. 2015;53(4):1115–20. PubMed Central PMCID: PMC4365258. 10.1128/JCM.02906-14 25609728 PMC4365258

[29] Masujin K, Orru CD, Miyazawa K, Groveman BR, Raymond LD, Hughson AG, et al Detection of Atypical H-Type Bovine Spongiform Encephalopathy and Discrimination of Bovine Prion Strains by Real-Time Quaking-Induced Conversion. Journal of clinical microbiology. 2016;54(3):676–86. PubMed Central PMCID: PMC4768002. 10.1128/JCM.02731-15 26739160 PMC4768002

[30] Orru CD, Groveman BR, Raymond LD, Hughson AG, Nonno R, Zou W, et al Bank Vole Prion Protein As an Apparently Universal Substrate for RT-QuIC-Based Detection and Discrimination of Prion Strains. PLoS Pathog. 2015;11(6):e1004983 PubMed Central PMCID: PMC4472236. 10.1371/journal.ppat.1004983 26086786 PMC4472236

[31] John TR, Schatzl HM, Gilch S. Early detection of chronic wasting disease prions in urine of pre-symptomatic deer by real-time quaking-induced conversion assay. Prion. 2013;7(3):253–8. PubMed Central PMCID: PMC3783112. 10.4161/pri.24430 23764839 PMC3783112

[32] Haley NJ, Carver S, Hoon-Hanks LL, Henderson DM, Davenport KA, Bunting E, et al Detection of chronic wasting disease in the lymph nodes of free-ranging cervids by real-time quaking-induced conversion. Journal of clinical microbiology. 2014;52(9):3237–43. PubMed Central PMCID: PMC4313144. 10.1128/JCM.01258-14 24958799 PMC4313144

[33] Davenport KA, Henderson DM, Mathiason CK, Hoover EA. Assessment of the PrPc Amino-Terminal Domain in Prion Species Barriers. J Virol. 2016;90(23):10752–61. 10.1128/JVI.01121-16 27654299 PMC5110164

[34] Cutlip RC, Miller JM, Race RE, Jenny AL, Katz JB, Lehmkuhl HD, et al Intracerebral transmission of scrapie to cattle. The Journal of infectious diseases. 1994;169(4):814–20. 8133096 10.1093/infdis/169.4.814

[35] Hamir AN, Miller JM, Kunkle RA, Hall SM, Richt JA. Susceptibility of cattle to first-passage intracerebral inoculation with chronic wasting disease agent from white-tailed deer. Veterinary pathology. 2007;44(4):487–93. 10.1354/vp.44-4-487 17606510

[36] Hamir AN, Kunkle RA, Miller JM, Bartz JC, Richt JA. First and second cattle passage of transmissible mink encephalopathy by intracerebral inoculation. Veterinary pathology. 2006;43(2):118–26. 10.1354/vp.43-2-118 16537929

[37] Biacabe AG, Morignat E, Vulin J, Calavas D, Baron TG. Atypical bovine spongiform encephalopathies, France, 2001–2007. Emerging infectious diseases. 2008;14(2):298–300. PubMed Central PMCID: PMC2600212. 10.3201/eid1402.071141 18258124 PMC2600212

[38] Greenlee MH, Smith JD, Platt EM, Juarez JR, Timms LL, Greenlee JJ. Changes in retinal function and morphology are early clinical signs of disease in cattle with bovine spongiform encephalopathy. PloS one. 2015;10(3):e0119431 PubMed Central PMCID: PMC4355414. 10.1371/journal.pone.0119431 25756286 PMC4355414

[39] Vrentas CE, Onstot S, Nicholson EM. A comparative analysis of rapid methods for purification and refolding of recombinant bovine prion protein. Protein Expr Purif. 2012;82(2):380–8. 10.1016/j.pep.2012.02.008 22381461

[40] Cheng K, Sloan A, Avery KM, Coulthart M, Carpenter M, Knox JD. Exploring physical and chemical factors influencing the properties of recombinant prion protein and the real-time quaking-induced conversion (RT-QuIC) assay. PloS one. 2014;9(1):e84812 PubMed Central PMCID: PMC3880330. 10.1371/journal.pone.0084812 24404191 PMC3880330

[41] Orru CD, Hughson AG, Groveman BR, Campbell KJ, Anson KJ, Manca M, et al Factors That Improve RT-QuIC Detection of Prion Seeding Activity. Viruses. 2016;8(5). PubMed Central PMCID: PMC4885095. 10.3390/v8050140 PMC4885095 27223300

[42] Orru CD, Groveman BR, Hughson AG, Zanusso G, Coulthart MB, Caughey B. Rapid and sensitive RT-QuIC detection of human Creutzfeldt-Jakob disease using cerebrospinal fluid. mBio. 2015;6(1). PubMed Central PMCID: PMC4313917. 10.1128/mBio.02451-14 PMC4313917 25604790

[43] Orru CD, Bongianni M, Tonoli G, Ferrari S, Hughson AG, Groveman BR, et al A test for Creutzfeldt-Jakob disease using nasal brushings. The New England journal of medicine. 2014;371(6):519–29. PubMed Central PMCID: PMC4186748. 10.1056/NEJMoa1315200 25099576 PMC4186748

[44] Richt JA, Hall SM. BSE case associated with prion protein gene mutation. PLoS Pathog. 2008;4(9):e1000156 PubMed Central PMCID: PMC2525843. 10.1371/journal.ppat.1000156 18787697 PMC2525843

[45] Nicholson EM, Brunelle BW, Richt JA, Kehrli ME Jr., Greenlee JJ. Identification of a heritable polymorphism in bovine PRNP associated with genetic transmissible spongiform encephalopathy: evidence of heritable BSE. PloS one. 2008;3(8):e2912 PubMed Central PMCID: PMC2488391. 10.1371/journal.pone.0002912 18698343 PMC2488391

[46] Heaton MP, Keele JW, Harhay GP, Richt JA, Koohmaraie M, Wheeler TL, et al Prevalence of the prion protein gene E211K variant in U.S. cattle. BMC veterinary research. 2008;4:25 PubMed Central PMCID: PMC2478677. 10.1186/1746-6148-4-25 18625065 PMC2478677

[47] Greenlee JJ, Smith JD, West Greenlee MH, Nicholson EM. Clinical and pathologic features of H-type bovine spongiform encephalopathy associated with E211K prion protein polymorphism. PloS one. 2012;7(6):e38678 PubMed Central PMCID: PMC3371052. 10.1371/journal.pone.0038678 22715405 PMC3371052

[48] Elder AM, Henderson DM, Nalls AV, Wilham JM, Caughey BW, Hoover EA, et al In vitro detection of prionemia in TSE-infected cervids and hamsters. PloS one. 2013;8(11):e80203 PubMed Central PMCID: PMC3815098. 10.1371/journal.pone.0080203 24224043 PMC3815098

[49] Zhang Y, Swietnicki W, Zagorski MG, Surewicz WK, Sonnichsen FD. Solution structure of the E200K variant of human prion protein. Implications for the mechanism of pathogenesis in familial prion diseases. J Biol Chem. 2000;275(43):33650–4. 10.1074/jbc.C000483200 10954699

